# Demographic patterns of walleye (*Sander vitreus*) reproductive success in a Wisconsin population

**DOI:** 10.1111/eva.13665

**Published:** 2024-03-10

**Authors:** Robert P. Davis, Levi M. Simmons, Stephanie L. Shaw, Greg G. Sass, Nicholas M. Sard, Daniel A. Isermann, Wesley A. Larson, Jared J. Homola

**Affiliations:** ^1^ Wisconsin Cooperative Fishery Research Unit University of Wisconsin‐Stevens Point Stevens Point Wisconsin USA; ^2^ Office of Applied Science, Wisconsin Department of Natural Resources Escanaba Lake Research Station Boulder Junction Wisconsin USA; ^3^ Department of Biological Sciences State University of New York‐Oswego Oswego New York USA; ^4^ U.S. Geological Survey, Wisconsin Cooperative Fishery Research Unit University of Wisconsin‐Stevens Point Stevens Point Wisconsin USA; ^5^ National Marine Fisheries Service, Alaska Fisheries Science Center, Auke Bay Laboratories National Oceanic and Atmospheric Administration Juneau Alaska USA

**Keywords:** parentage analysis, phenotypic traits, recruitment, walleye

## Abstract

Harvest in walleye *Sander vitreus* fisheries is size‐selective and could influence phenotypic traits of spawners; however, contributions of individual spawners to recruitment are unknown. We used parentage analyses using single nucleotide polymorphisms to test whether parental traits were related to the probability of offspring survival in Escanaba Lake, Wisconsin. From 2017 to 2020, 1339 adults and 1138 juveniles were genotyped and 66% of the offspring were assigned to at least one parent. Logistic regression indicated the probability of reproductive success (survival of age‐0 to first fall) was positively (but weakly) related to total length and growth rate in females, but not age. No traits analyzed were related to reproductive success for males. Our analysis identified the model with the predictors' growth rate and year for females and the models with year and age and year for males as the most likely models to explain variation in reproductive success. Our findings indicate that interannual variation (i.e., environmental conditions) likely plays a key role in determining the probability of reproductive success in this population and provide limited support that female age, length, and growth rate influence recruitment.

## 1 INTRODUCTION

Fishing mortality is a size‐selective process that can influence phenotypic variation within exploited fish populations, and consequently demographics (Hidalgo et al., 2011; Matsumura et al., 2011; Sinclair et al., 2002), which could influence recruitment potential (Shaw et al., 2018). Fishery managers attempt to influence recruitment potential in exploited fish populations by implementing regulations such as length limits, bag limits, and seasonal fishing moratoriums (Ahrens, 2021; Arendse et al., 2007; Post, 2013; Rola et al., 2018). Harvest regulations may induce selective pressure by protecting population segments from harvest, possibly decreasing trait variation, and therefore potentially diminishing a population's resiliency to environmental change (Hard et al., 2008; Lewin et al., 2006; Wright & Trippel, 2009). Potentially adaptive traits that may experience harvest‐induced selection may include physical traits such as coloration, body morphology, age at maturation, and growth rate (Mousseau & Roff, 1987; Schaefer et al., 2015; Vandeputte et al., 2004) or behavioral traits such as the timing of spawning, spawning migration, and habitat selection (Carscadden et al., 1997; Leclerc et al., 2017; Oomen & Hutchings, 2015). Identifying the effects of selection and the traits of individuals that successfully contribute to successive generations is important to understanding the influence of fishing mortality and management actions on population sustainability.

Parental (specifically maternal) effects have been shown to play a role in stock‐recruitment dynamics by influencing the quantity and quality of eggs and larvae produced by a spawning population (Fuiman & Ojanguren, 2011; Hixon et al., 2014; Pepin & Myers, 1991). Maternal effects are defined as non‐genetic influences of adult females on offspring survival (Green, 2008). Female fecundity is associated with fish length in many species (e.g., Morita & Takashima, 1998; Serns, 1982), and therefore, the size structure of the female spawning stock may play a role in population resiliency (Shaw et al., 2018; Venturelli et al., 2010). Despite what may be considered a theoretically intuitive relationship between the number of spawning individuals and recruitment (i.e., juveniles cannot be produced without spawning adults), stock‐recruitment relationships have been difficult to demonstrate in practice (Myers, 2001), at least in part due to life history characteristics of fishes that are of economic or recreational importance (Winemiller, 2005). Environmental conditions have been a more reliable but still variable predictor of recruitment in some cases (Myers, 1998), even when standing stock biomass has not (e.g., Beard et al., 2003). In contrast, maternal effects related to fecundity and female size structure have been linked to recruitment (Hixon et al., 2014; Scott et al., 2006), in some cases more strongly than measures of spawning stock biomass (Shaw et al., 2018). Conventional wisdom among many fisheries biologists and the general public is that longer females contribute a relatively greater number of offspring to a cohort, which is supported to some degree by research (Andersen et al., 2019; Barneche et al., 2018; Hixon et al., 2014). This has been aptly named the “Big Old Fat Fecund Female Fish” (BOFFFF) hypothesis (Hixon et al., 2014). However, limited capacity to link offspring to their respective parents' past early life history stages has made it difficult to test whether individual fish or segments of a population disproportionately contribute to recruitment measured at life stages past egg or larval juveniles.

Genetic tools provide a means of identifying parent‐offspring relationships in natural settings (e.g., Andersson et al., 2017; Baetscher et al., 2018; Hohenlohe et al., 2018). High‐throughput genotyping panels allow high‐resolution genetic data to be efficiently collected at hundreds to thousands of single nucleotide polymorphism (SNP) loci (Meek & Larson, 2019), enabling large‐scale pedigrees to be reconstructed in wild populations (Sard et al., 2020; Weise et al., 2022). The process for developing an effective SNP panel has been detailed by Bootsma et al. (2020), which provided a genotyping‐in‐thousands panel (GT‐seq; Campbell et al., 2015) of 429 loci capable of genetic stock identification and parentage analysis for walleye *Sander vitreus* in Wisconsin.

Walleye are a culturally (Native American and First Nations subsistence), economically, and recreationally important game species throughout the United States and Canada that supports harvest‐oriented fisheries in many areas (Erickson et al., 2019; Gaeta et al., 2013; Mrnak et al., 2018; Tufts et al., 2015). Despite harvest and catch rates being hyperstable (Mosley et al., 2022; Mrnak et al., 2018), there is some evidence of declining productivity and natural recruitment of walleye fisheries in the Ceded Territory of Wisconsin (Embke et al., 2019; Mrnak et al., 2018; Rypel et al., 2018). Declines in walleye populations are predicted across the upper Midwest, at least in part due to climate change and associated shifts towards centrarchid‐dominated fish assemblages (Hansen et al., 2018). Another potential contributor to declining natural recruitment is the high mortality of age‐0 walleye between the time of hatching and the following fall, which has been identified as a period in walleye life history where a recruitment bottleneck may occur (Gostiaux et al., 2022). It remains unknown whether offspring of parents with variable traits may show differential survival through this and other potential recruitment bottlenecks.

Walleye are iteroparous broadcast spawners that spawn in spring following ice‐out on rock and gravel habitats in lakes and rivers and provide no parental care to eggs or offspring (Bozek et al., 2011). Walleye exhibit sexual dimorphism, as females tend to grow to longer lengths and mature 1–2 years later than males (Bozek et al., 2011; Henderson et al., 2003). Walleye fecundity (Johnston & Leggett, 2002; Serns, 1982) and egg size (Johnston & Leggett, 2002) have been correlated to female length over broad geographic scales and latitudinal (i.e., temperature) gradients (Johnston & Leggett, 2002). Feiner et al. (2016) found that egg size increased and variation in egg size decreased with increasing female length across several walleye populations in the Great Lakes region. Egg weight and hatching success have also been associated with the size and age of the spawning female (Johnston, 1997). Additionally, the length of walleye larvae after hatching was positively related to egg size, indicating that walleye fry from larger eggs, typically from larger females, may have an early survival advantage (Johnston, 1997). A positive relationship between egg size and larval length has also been observed in yellow perch *Perca flavescens* (Andree et al., 2015). Conversely, Johnston et al. (2021) found that embryonic survival was more strongly tied to egg properties such as size and fatty acid composition and not to maternal characteristics such as length or age. In the absence of strong maternal effects within populations, egg characteristics can be linked to environmental factors such as productivity (Wang et al., 2012).

Although there are examples of paternal effects in some fish species, including broadcast spawners (Kamler, 2005; Neff, 2004; Politis et al., 2017), investigations of paternal effects in walleye have been limited to sperm quality in wild populations (Casselman et al., 2006), or to improving hatchery rearing techniques (e.g., Rinchard et al., 2005). Individual‐level disparities in egg quantity and quality that translate to disparities in the fitness of larvae reflect potential maternal effects that may translate to population‐level changes in recruitment, especially under the influence of exploitation (Venturelli et al., 2010). For example, in Escanaba Lake accounting for size structure of the females in the population by relating recruitment to overall egg production explained more variation in recruitment than simply estimating adult stock abundance or female relative abundance (Shaw et al., 2018). Given the potential for parental effects in walleye and declining natural recruitment and productivity in some populations, testing whether phenotypic traits and population demographics influence walleye recruitment would be informative.

We used genetic‐based parentage analysis using SNPs to better understand basic walleye reproductive outcomes that have not been described previously (e.g., number of mates, number of offspring per parent) in Escanaba Lake, Wisconsin, USA during 2017–2020. Specifically, we tested whether parental traits such as total length (TL), age, and growth rate influenced the probability age‐0 survival to the first fall. We hypothesized that age‐0 survival would be related to TL, age, and growth rate of parents and predicted that the probability of producing an offspring would be higher for (1) faster‐growing fish; (2) longer fish, and (3) older fish. We assumed that faster‐growing fish were the most fit and therefore would have the highest reproductive success, while previous research has shown relationships between length and age and reproductive output (Johnston, 1997; Johnston & Leggett, 2002). We also hypothesized that interannual variation would influence the likelihood of parental success. We conducted our multi‐year assessment using the walleye population in northern Wisconsin's Escanaba Lake, a system that is sampled annually to index the adult population during spring spawning and the age‐0 population in the fall.

## 2 METHODS

### 2.1 Study area

Escanaba Lake is a 119‐ha mesotrophic drainage lake in the Northern Highland Fishery Research Area of Vilas County, Wisconsin, with a maximum depth of about 8 m (refer to Sass et al., 2022 for more detail). The riparian zone of Escanaba Lake is nearly undeveloped (>99%) and the lake has supported a naturally reproducing walleye population since the late 1950s when walleye were initially stocked (Sass et al., 2022). Besides walleye, the fish assemblage includes naturally reproducing populations of muskellunge *Esox masquinongy*, northern pike *Esox lucius*, smallmouth bass *Micropterus dolomieu*, yellow perch, bluegill *Lepomis macrochirus*, pumpkinseed *Lepomis gibbosus*, rock bass *Ambloplites rupestris*, white sucker *Catostomus commersonii*, bluntnose minnow *Pimephales notatus*, and common shiner *Luxilus cornutus*. Since 1946, Escanaba Lake has been used as an experimental lake to test for species‐specific harvest regulations (Patterson, 1953) and has maintained a compulsory creel census of all anglers fishing the lake (Shaw et al., 2021). Experimental regulations for walleye on Escanaba Lake originated with no regulation on length and harvest (1946–2003) and were changed to a 71‐cm minimum length limit with a daily bag limit of one fish in 2003 (Hansen et al., 2011), which was maintained through the 2021 fishing season. Essentially no legal harvest of recreational angler‐caught walleye occurred during the 71‐cm minimum length limit regulation period (Haglund et al., 2016).

### 2.2 Sample collection

Genetic sample collection was performed concurrently with annual spring (adult) and fall (age‐0) mark‐recapture walleye population estimates performed by the Wisconsin Department of Natural Resources (WDNR) (refer to Shaw et al., 2018 for detailed description). Eight fyke nets deployed at known spawning locations throughout the lake were used to collect and mark (temporary fin clip) adult walleye (i.e., sexed through the expression of gametes, ≥381 mm) shortly after ice‐out in the spring from 2017 to 2019. Walleye were removed from nets daily throughout the duration of spawning, which can last from 1 to 4 weeks. A single electrofishing run during the peak of spawning was used to recapture marked individuals to estimate adult population abundance. Genetic material was collected from all walleye sampled during the spring fyke netting and the electrofishing survey. Fall sampling (September–October) for age‐0 and age‐1 walleye consisted of mark‐recapture surveys using boat electrofishing. A survey consisted of one run of the entire shoreline of the lake in one night. Surveys were repeated, once per week, until a mark‐recapture population estimate with reasonable confidence was achieved (i.e., coefficient of variation <40%; Shaw et al., 2018). Genetic samples were collected from all age‐0 and age‐1 walleye sampled on each electrofishing run. Due to the COVID‐19 pandemic in 2020, only fall electrofishing occurred. Length, weight (for a subset of fish by sex in each year), identifying marks (i.e., t‐bar anchor tags or passive integrated transponder tag numbers), dorsal spines and/or scales for age estimation, and a tissue sample (fin clip preserved in ethanol) were collected for each fish. Dorsal spines were used as the primary structure for estimating age for adult walleye collected in the spring, while scales were used to develop age‐length cutoffs for age‐0 and age‐1 fish collected in fall. For unknown sex fish (no visible expression of gametes), sex could be assigned from a previous survey observation if it had been sampled in multiple years. We used sex‐specific length‐at‐age rank percentiles based on the distribution of all TLs for walleye of a specific sex and age collected from Escanaba Lake in the preceding 10 years to categorize growth for each adult fish.

### 2.3 Laboratory methods

DNA was extracted using the chelating resin approach described by Walsh et al. (1991). Each sample was immersed in a solution containing 10% chelating resin (Bio‐Rad Chelex‐100), 98% exonuclease‐free water, 1% NP‐40 detergent (Thermo Fischer Scientific, Fluka, catalog number: 74358), and 1% Tween‐20 then incubated at 99°C for 10 min. We then used the GT‐seq approach (Campbell et al., 2015) to genotype a panel capable of pedigree analysis targeting 429 loci containing one or more SNPs (Bootsma et al., 2020). One negative control consisting of sterile water in place of template DNA was included in each 96‐well plate. Genotyping methods are described in (Bootsma et al., 2020). A total of 2763 samples were prepared for sequencing, and sequencing was performed by the University of Wisconsin Biotechnical Center using three flowcells (Illumina NovaSeq SP with 300 cycle chemistry).

### 2.4 Genomic data filtering

Quality control and initial filtering of genomic data occurred in five stages. First, all SNPs that genotyped in <80% of samples were removed. Second, inbreeding coefficient values (*F*_IS_) were calculated for each SNP and a SNP was removed if the absolute value exceeded 0.08, with the cutoff conservatively positioned to remove distributional outliers (Shafer et al., 2017). *F*_IS_ values are based on the ratio of observed and expected heterozygosity and can be useful for indicating inbreeding. Excessive *F*_IS_ values can be caused by the presence of null alleles and homologous sequences, which may result in incorrect genotypes (Waples, 2015). Next, samples with genotyping rates <80% were removed and individuals with a contamination score >0.4 were removed (GTscore; McKinney et al., 2019). We also identified duplicate samples as individuals with identical genotypes at >90% of loci and retained one individual at random from each set of duplicates. Finally, we thinned the genotypic data to diminish the potential influence of linkage by first removing all but one SNP per amplicon, then removing one SNP per pair with a standardized index of association (Agapow & Burt, 2001) value >0.25.

### 2.5 Parent‐offspring assignments

To assign offspring to year classes during 2017–2020, walleye ≤190 mm TL were assumed to be age‐0 based on the age‐length distributions of fish (Figure S1). Age‐length distributions did not provide a clear distinction between age‐1 and age‐2 fish, but a TL of 275 mm was used as a conservative upper boundary for delineating age‐1 walleye (only 5% of walleye >275 mm were age‐1), which likely excluded extremely fast‐growing individuals in this age category. Consequently, age‐1 fish were only used to assess the accuracy of assigning parent‐offspring pairs in a specific year. Assignment of parents to age‐1 fish was used to evaluate sampling effort across years, where low overlap between individuals identified as parents with age‐0 and age‐1 offspring would indicate a large proportion of unidentified parents in the previous year when offspring of a specific year class were age‐0.

Parent‐offspring assignments were made using the program Colony (Jones & Wang, 2010). Our configuration of Colony implemented a maximum‐likelihood algorithm to infer sibships and parentage jointly over the entire pedigree configuration by iteratively comparing alternate possible configurations (Jones & Wang, 2010). Separate parentage analyses were performed for each year of collection, with all candidate parents included, regardless of the year the potential parent was sampled. We parameterized Colony by selecting a medium‐length run with no sibship prior, and medium precision. Although inbreeding was unlikely, the option of inbreeding being a possibility was included in the analyses. Individuals of unknown sex (*n* = 58) were included in both the male and female parental datasets. There were no parent assignments with two parents of unknown sex. Parent‐offspring relationships were accepted if the probability of assignment exceeded 90%. For the purposes of estimating the number of mates by sex in each year, we included unsampled parents with distinct genotypes inferred by Colony. For all other analyses, only individuals sampled were included.

Pedigree accumulation analysis (Sard et al., 2021) was used to estimate the number of spawning individuals (*N*_s_) that contributed to each sampled cohort. Pedigree accumulation analysis generates curves that are similar to species accumulation curves used to estimate species diversity (Ugland et al., 2003). The function “specaccum” in the R package “vegan” (Oksanen et al., 2022) was used to generate the pedigree accumulation curves, and the vegan function “specpool” was used to estimate the total number of parental genotypes for each annual cohort using the Chao (1987) and jackknife methods (Heltshe & Forrester, 1983). We performed a simulation to test for potential biases of the generated N_s_ estimates (refer to Supplementary Information for methods and results).

### 2.6 Statistical analysis

We filtered our adult walleye dataset to account for uncertainties in mortality and reproductive maturity for certain individuals. Mortality of adult fish after they are encountered could result in an overestimation bias for the proportion of fish that did not produce offspring, as these fish may not be alive to reproduce in subsequent spawning seasons. Therefore, we incorporated mortality into our analyses by first generating a matrix for each adult walleye that was composed of four unique year × individual combinations (i.e., four rows of data) that reflected whether each fish was known to be alive or not. A fish was considered to be alive for a given spawning season if it was sampled or identified as a parent in that year or a subsequent year. Years where fish were known to be alive were retained throughout all analyses. The long‐term natural mortality rate for Escanaba Lake (19.8%, Nate et al., 2011) was applied at random to adult walleye in each year sequentially in which an individual had an unknown fate to correct for individuals that were unable to reproduce due to their preceding death. Because harvest was essentially prohibited on Escanaba Lake during our study, fishing mortality (including hooking mortality) was assumed to be zero (Haglund et al., 2016; Sass et al., 2022). We then performed analyses iteratively to account for the random removal of individuals of unknown fate to find an “average” relationship among reproductive success and the independent variables being evaluated. Only age‐3+ walleye were included in our analysis to avoid inclusion of juveniles (Bozek et al., 2011).

We tested the hypothesis that reproductive success was a function of parental traits (age, TL, growth rate) using logistic regression. Reproductive success for a parent was coded as 1 if at least one age‐0 offspring from the parent was detected in that year; parents with no offspring detected were coded as 0 in a given year. The effects of TL, age, and growth rate of individual parents on the probability of producing at least one offspring were evaluated separately with sex‐specific univariate models. When estimating the relationship between total length and reproductive success, only individual fish × year combinations where the fish were handled and measured were included because TL of individual fish cannot be accurately predicted in years the fish was not handled. Parental age can be inferred from the point at which age was estimated (e.g., a 5‐year‐old fish is 7 years old 2 years later). Rank percentiles of TL at age (henceforth, growth rate) were considered constant over time. For example, if a fish was at the 75th percentile of growth at age 5, we assumed it was at this percentile at all ages. Hence, age and growth rank percentiles could be analyzed for all 4 years after accounting for natural mortality, which was accomplished using 1000 iterations for each model by randomly removing individuals each year of the study to simulate deaths before running the analysis. Median model coefficients and diagnostic statistics were then calculated. As a measure of variability, the median standard error was reported. Nagelkerke's *R* ^2^ is presented to assess model fit (Norman & Streiner, 2014). The odds ratio of the regression coefficients is presented as a measure of effect size, along with the 95% confidence interval. Significant relationships were determined by the median p value for the slope of the regression, with *α* < 0.05 to denote statistical significance.

Next, we used a two‐step model selection procedure to assess the relative importance of age, growth rate percentile, and interannual variation (denoted as the variable “year”) on mortality‐corrected reproductive success using a general linear model with a binomial distribution. TL was not considered as part of the candidate models because it could not be estimated across multiple years. First, we tested for the most likely model selected from a list of candidate models. The candidate models included:

Model 1: Produced Offspring ~ Null
Model 2: Produced Offspring ~ Age
Model 3: Produced Offspring ~ Year
Model 4: Produced Offspring ~ Growth Rate Percentile
Model 5: Produced Offspring ~ Age + Growth Rate
Model 6: Produced Offspring ~ Age + Year
Model 7: Produced Offspring ~ Growth Rate Percentile + Year
Model 8: Produced Offspring ~ Growth Rate Percentile + Age + Year

For each sex, the best candidate model was selected as having the lowest Bayes Information Criterion (BIC) of the candidate models in each iteration over 1000 iterations. The model that was determined to be the top model across each iteration was tallied, and the model with the most counts was considered to be the best candidate model. BIC weights of the model are also presented. After the most likely candidate model was selected, a second run of 1000 iterations was conducted with the selected model to obtain the median model coefficients and statistics. The same suite of diagnostic statistics was obtained as with the previous univariate logistic regression models. All analyses except where explicitly stated were conducted in R version 4.1.1 (R Core Team, 2021), including the use of the following packages; “tidyverse” (Wickham et al., 2019), “ggVennDiagram” (Gao, 2022), “cvAUC” (LeDell & Petersen, 2022), “ape” (Paradis & Schliep, 2019), “adegenet” (Jombart, 2008), “poppr” (Kamvar et al., 2014), and “pegas” (Paradis, 2010).

## 3 RESULTS

A total of 2477 of the 2763 (90%) walleye sequenced provided data that passed all quality control filters. Of the 286 samples that were removed, 44 genotyped poorly (<80% genotyping rate), 68 demonstrated risk of contamination as evidenced by elevated rates of heterozygosity (McKinney et al., 2019), and 184 were duplicates. The duplicated samples were largely attributed to younger fish that were below the minimum length threshold to receive a uniquely numbered t‐bar anchor tag or had shed the tag among years. None of the negative controls yielded sequences that could be genotyped. After filtering, 701 SNPs were retained across 371 loci. Of the 352 SNPs removed, 90 were genotyped in <80% of samples and 272 were removed due to excessive *F*_IS_ values. Following thinning for linkage, the final dataset consisted of 299 SNPs.

A total of 535 age‐0 walleye were successfully assigned to at least one parent, and 397 (191 female and 206 male) adult walleye were assigned as a parent, representing 79.3% and 29.6% of the total individuals genotyped, respectively (Table 1). Maternal assignments of age‐0 offspring averaged 43% across years and paternal assignment averaged 41%, but assignment rates were variable year to year. Assignments of both parents occurred for 18% of offspring. Age‐1 walleye maternal assignments occurred for an average of 43% of screened individuals across years and paternal assignments occurred at an average rate of 39%. The overlap of parents (i.e., identical parents detected between age‐1 and age‐0 fish the previous year) detected among years was 14.5% (range = 11%–20%; Figure S2). Throughout the duration of the study, no offspring were detected for about 38% of the genotyped adult walleye. The maximum number of offspring detected for a single parent across all 4 years was 12 (Table S2), a female with seven offspring detected in 2019, four in 2018, and one during 2017. The average number of offspring per parent was similar across all 4 years of the study (1.27–1.76 for females, 1.11–1.43 for males). The number of offspring per individual for males and females had more variation in 2018 compared to other years (Table S3).

**TABLE 1:** Life stage, year, number of offspring, number and proportion of mothers and fathers assigned to offspring, and number and proportion of total parental assignments made using Colony.

Life stage	Year	Potential offspring	Mother (%)	Father (%)	Zero parents (%)	One parent (%)	Both parents (%)
Age‐0	2017	135	65 (48)	50 (37)	44 (33)	91 (67)	24 (18)
Age‐0	2018	419	183 (44)	151 (36)	151 (36)	268 (64)	66 (16)
Age‐0	2019	130	51 (39)	49 (38)	49 (38)	81 (62)	19 (15)
Age‐0	2020	134	56 (42)	70 (52)	39 (29)	95 (71)	31 (23)
Age‐1	2018	86	38 (44)	35 (41)	24 (28)	62 (72)	11 (13)
Age‐1	2019	93	39 (42)	30 (32)	37 (40)	56 (60)	13 (14)
Age‐1	2020	73	32 (44)	32 (44)	24 (33)	49 (67)	15 (21)

**FIGURE 1:**
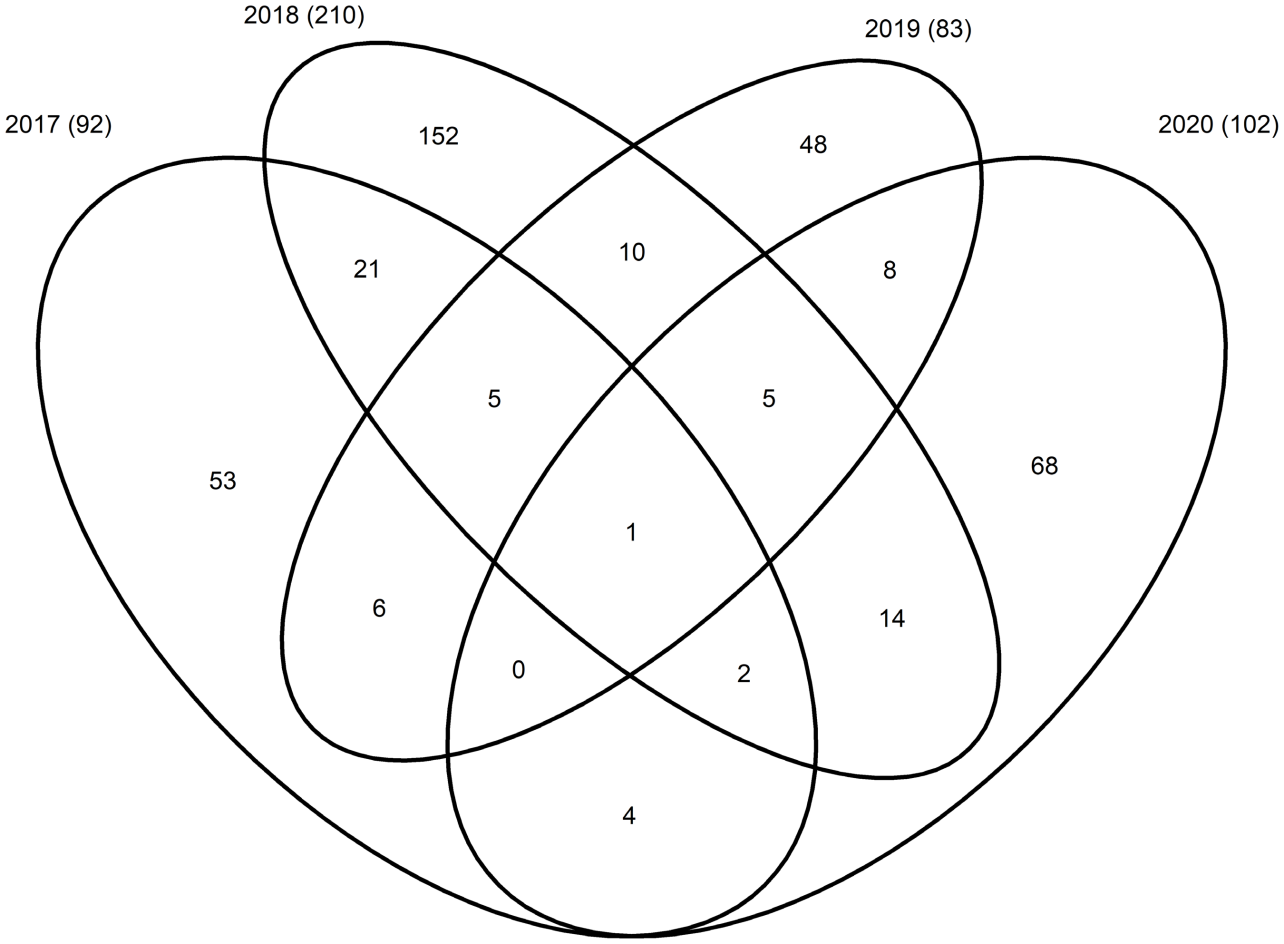
A Venn diagram of the number of parents with detected offspring in each year juveniles were sampled in Escanaba Lake. The number in parentheses represents the total number of parents identified in each year.

For subsequent analyses, only age‐0 offspring and 1339 adults were used to test for the relationships between reproductive success and parental traits, with subsets being used after various filters were applied (refer to Table S4 for summary statistics of parents). Of these individuals, sex was known for 1281 parents based on visual expression of gametes or assignment during the parentage analysis, permitting their inclusion in sex‐specific statistical analyses. Females sampled were on average longer than males sampled in the same year and tended to be older, except in 2019 when males and females were of similar age. Age‐0 offspring were detected most often in only a single year for a given parent, with only one individual having age‐0 offspring detected in all 4 years (Figure 1). Altogether, 89% of females and 91% of males identified as parents were assigned to 1 or 2 juveniles. The highest number of juveniles assigned to a parent in a given year was 7 for both sexes.

**FIGURE 2:**
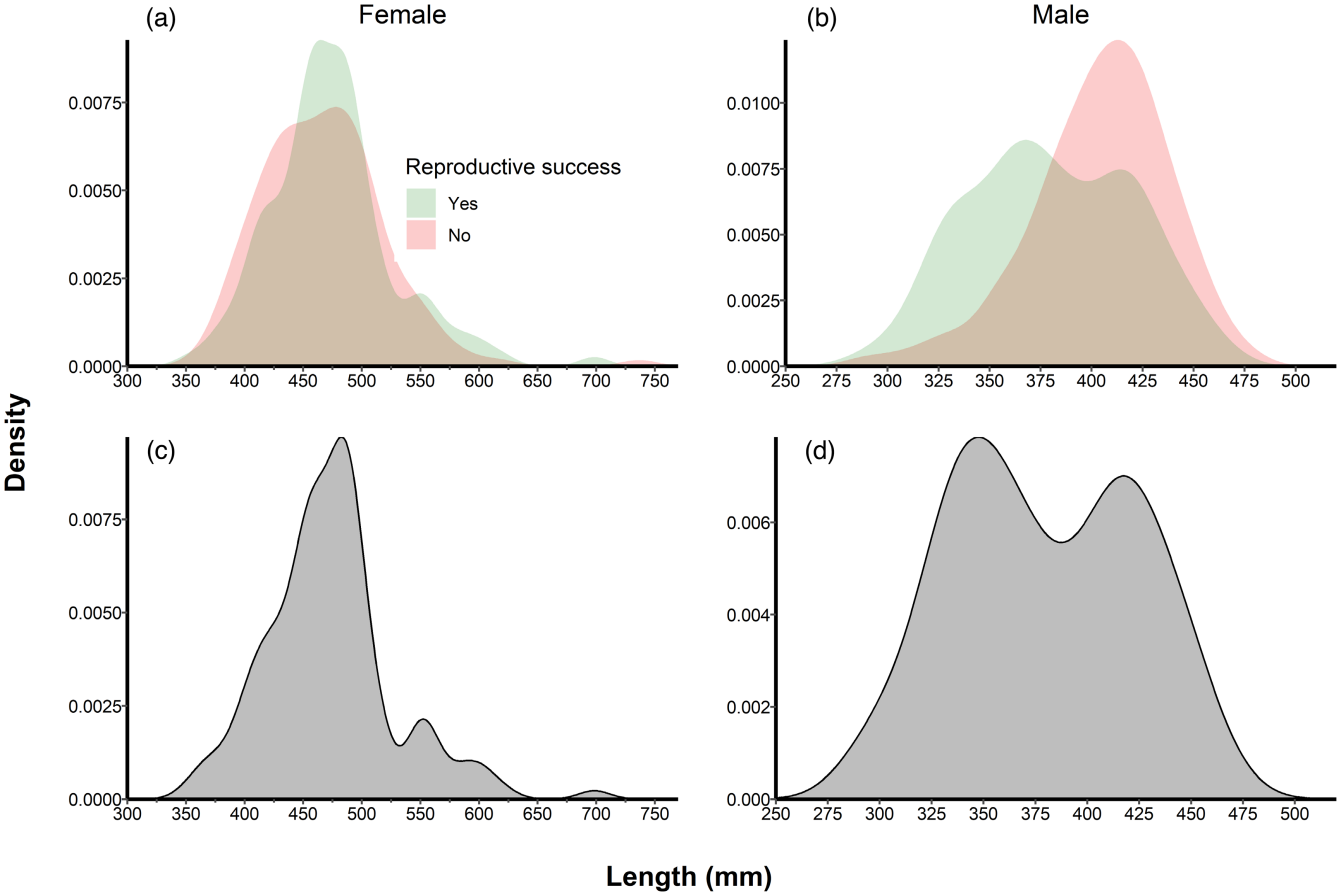
Density plots of the total length (mm) of the distribution of successful (green) and unsuccessful (red) female walleye *Sander vitreus* and male walleye parents (a and b, respectively) and the distribution of the number of offspring produced by walleye parents at a given length for females and males (c and d, respectively).

Excluding males in 2019 (Figure S3f), age‐TL distributions of the adults sampled were relatively consistent across years. Males identified as parents tended to be shorter than males that were not identified as parents (Figure 2b) and most females identified as parents occurred in a relatively narrow TL range of 400–500 mm (Figure 2a). The distribution of the number of offspring produced in relation to TL was nearly identical to the full TL distribution of both males and females (Figure 2d,c, respectively) indicating the number of offspring detected for a given TL generally reflected the number of adults at that length. The median age of females identified as parents was 9, and the median age of females where no offspring were detected was 8 (Figure S4). The median age of males identified as parents was 5, and the average age of males where no offspring were detected was 8, although the distribution of ages for both groups of males was similar after accounting for potential mortality (Figure S4d).

**FIGURE 3:**
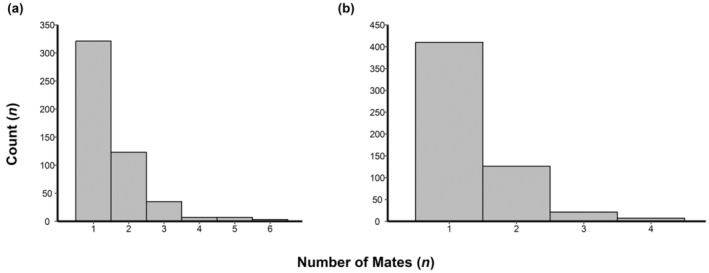
The number of detected mates for females (a) and males (b) in a given year in this study.

Successfully reproducing female adult walleye had an average of 1.44 mates per year across all years, with 64% of females having one mate and 90% of females having two or fewer mates (Figure 3, Table 2). Females in 2018 (1.75 mates/female) had more mates on average than the other 3 years (1.33–1.40 mates/female). In 2018, females had a maximum of six mates (three occurrences) whereas no female had more than three mates in the other 3 years of the study. Males had slightly fewer mates per individual with an average of 1.28 mates among all years, with 2018 again having more mates per individual (1.49 mates/male) than other years (1.15–1.26 mates/male). Like females, the maximum number of observed mates was also greater during 2018 (seven individuals with four mates) than other years (two or three mates). In males, 73% that produced an offspring had one mate and 95% had two or fewer mates.

**TABLE 2:** Number of mates per parent and standard deviation (SD) in each year across the study.

Year	Females		Male	
	Mean	SD	Mean	SD
2017	1.38	0.62	1.26	0.54
2018	1.75	1.11	1.49	0.73
2019	1.34	0.59	1.23	0.45
Mean across years	1.44	0.72	1.28	0.52

**FIGURE 4:**
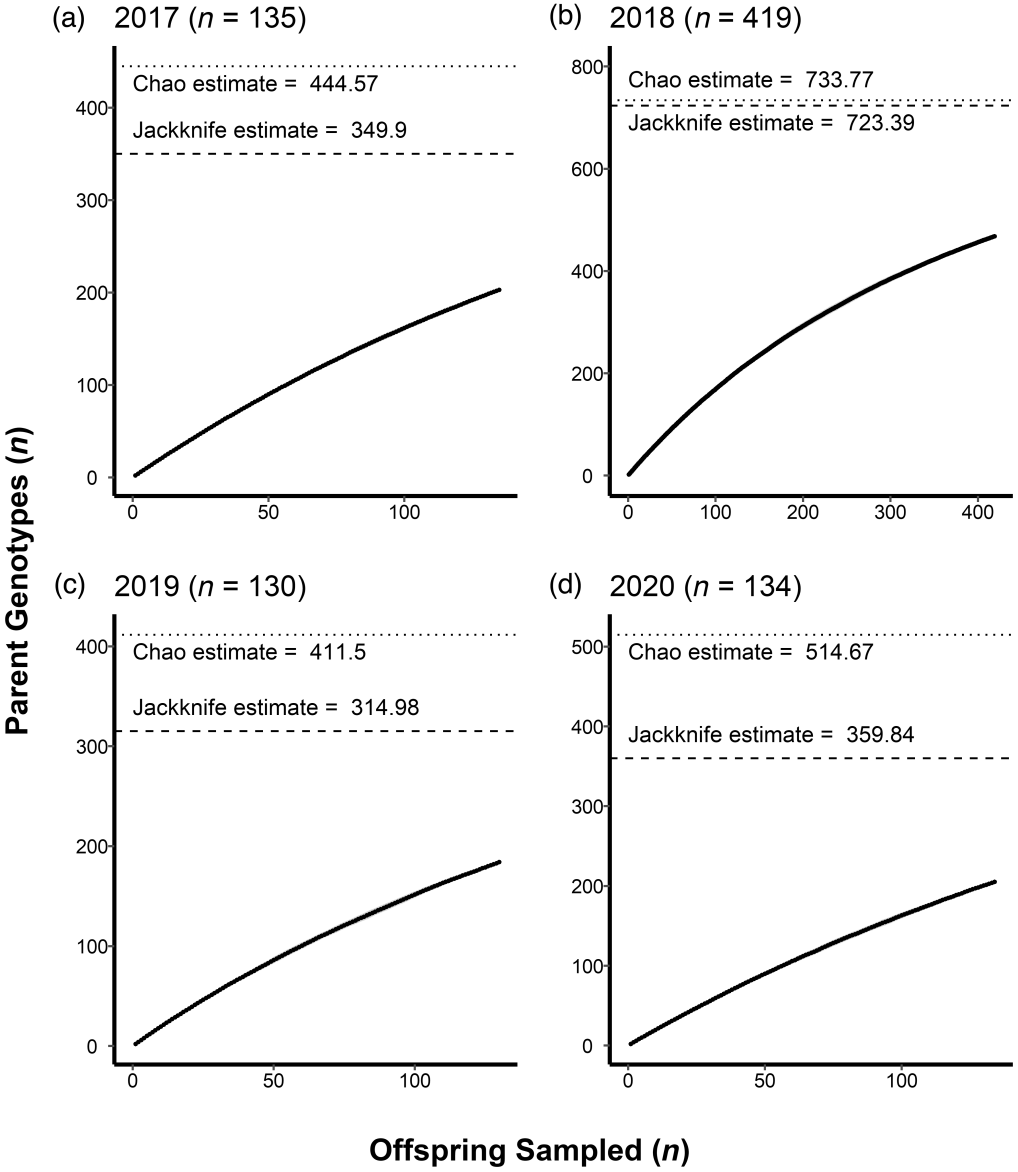
Parent accumulation plots for Escanaba Lake in 2017–2020. Dotted lines represent the Chao estimate for the number of successfully reproducing adults, and dashed lines represent the jackknife estimate.

More individuals produced offspring successfully in 2018 when compared to other years in this study (Figure 4). The Chao estimates (± std. error) of the number of successfully spawning individuals derived from pedigree accumulation analysis during 2017 to 2020 are 445 (±55.8), 73 (±44.1), 441.5 (±56.2), and 514.7 (±72.3), respectively. The jackknife estimates for the number of successful spawners are 349.9 (±14.9), 723.4 (±18.5), 315.0 (±14.4), and 359.8 (±16.1) for 2017 to 2020, respectively.

**FIGURE 5:**
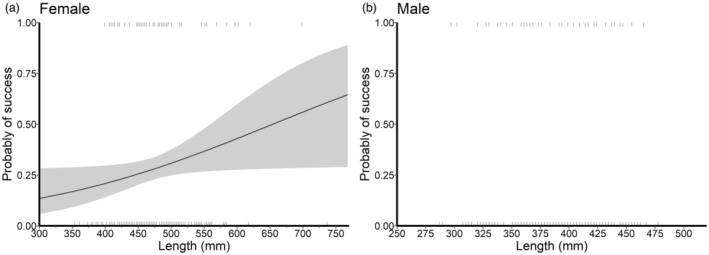
The relationship between length of parent and the probability of reproductive success in 1 year for female and male walleye *Sander vitreus* (a and b, respectively) with the 95% confidence interval in the shaded region. Dashes represent individuals who successfully reproduced (top) and did not reproduce (bottom). No significant relationship was detected for male walleye.

**FIGURE 6:**
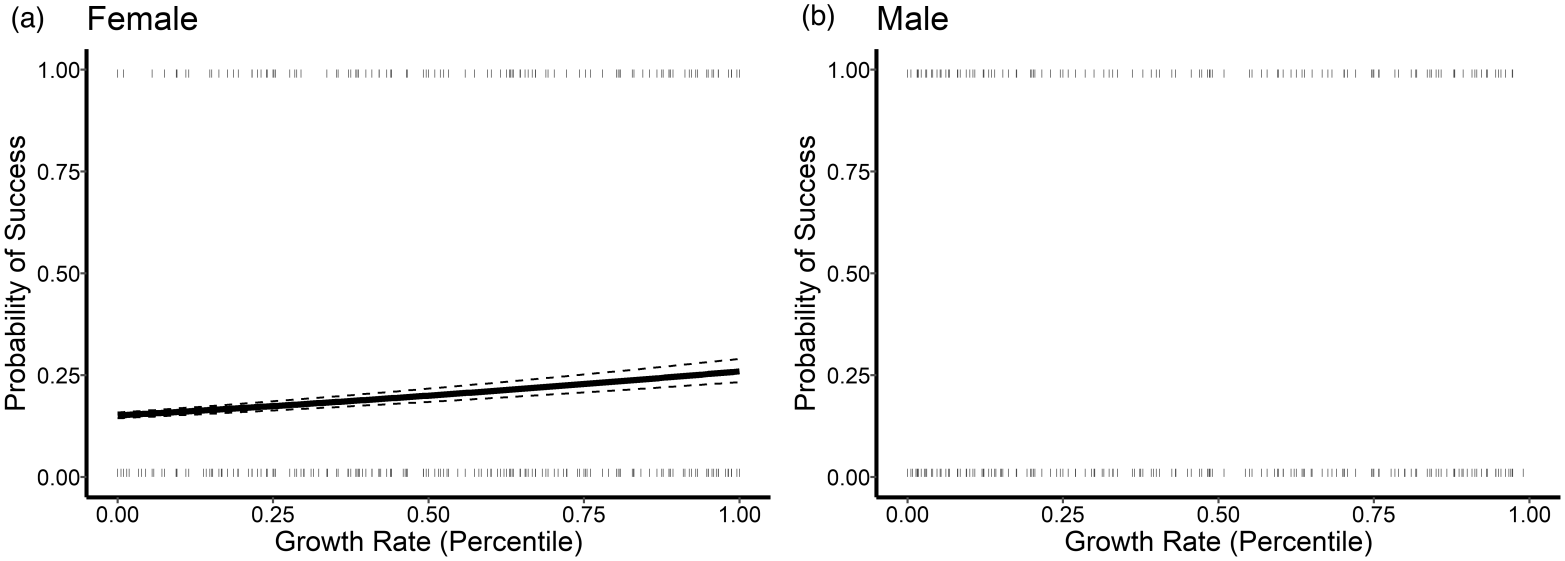
The relationship between growth rate and the probability of reproduction success (solid line) in 1 year for female and male walleye *Sander vitreus* (a and b, respectively) with the lines with slopes and intercepts at the 2.5 and 97.5 percentiles (dashed lines) after the iterative mortality correction (*i* = 1000 iterations). Dashes represent individuals who successfully reproduced (top) and did not reproduce (bottom). No significant relationship was detected for male walleye.

In females, reproductive success was positively related to TL (Table 3). The probability of producing an offspring for female walleye increased about twofold from 360 mm in TL (~15%), roughly the length when female walleye can begin to reproduce, to 530 mm in TL (~34%; Figure 5). Male reproductive success was not related to TL. In both cases, the models had relatively high accuracy, but low predictive power and relatively low pseudo *R* ^2^ (Table 3) due to the high number of unsuccessful parents in the dataset. For female walleye, reproductive success was positively related to growth rate percentile (Table 4), with the median probability of producing an offspring increasing from 17% at the 25th percentile to 20% at the 50th percentile and 23% at the 75th percentile (Figure 6a). There was no relationship between reproductive success of male walleye and growth rate percentile. The age of parents was not a significant predictor of reproductive success for either sex (Table 4; Figure S4).

**TABLE 3:** A summary of the logistic regression models for length and by sex on the probability of reproductive success.

Model	Sex	Intercept	SE	Slope	SE	OR_slope_	CI_OR_	*R* ^2^
Length	Female	−3.436	1.232	0.005*	0.003	1.0053	1.0002–1.0105	0.023
Length	Male	0.622	1.55	−0.0053	0.004	0.995	0.987–1.002	0.011

*Note*: Significance of regression coefficients (Intercept and slope) is represented as follows: *p < 0.05, **p < 0.01, and ***p < 0.001.

Abbreviations: CI_OR_, 95% confidence interval of the odds ratio of the slope; OR_slope_, odds ratio of the slope based on a 1 mm change; *R* ^2^, Nagelkerkes *R* ^2^; SE, standard error.

**TABLE 4:** Median values for the model coefficients and diagnostics from 1000 model iterations with mortality incorporated.

Model	Sex	Intercept	SE_int_	Slope	SE_slope_	OR_slope_	CI_OR_	*R* ^2^	*n*
Growth Rate	F	−1.73	0.17	0.676	0.283	1.966	1.129–3.424	0.01	944
Growth Rate	M	−1.11	0.14	−0.298	0.257	0.742	0.449–1.228	0.002	957
Age	F	−1.56	0.235	0.021	0.025	1.021	0.972–1.073	0.001	947
Age	M	−1	0.203	−0.036	0.028	0.965	0.913–1.019	0.003	963

Abbreviations: CI_OR_, 95% confidence interval of the odds ratio of the slope; OR_slope_, odds ratio of the slope based on a 1 mm change; *R* ^2^, Nagelkerkes *R* ^2^; SE, standard error.

For females, model 7 with the predictor's growth rate and year was selected as the mostly likely model based on BIC in every iteration. For males, model 3 (reproductive success ~ year) and model 6 (reproductive success ~ age + year) were selected in 54% and 46%, respectively, of the iterations as the most likely model. In model 3, males had a statistically greater probability of reproducing in 2018 compared to the other years in the study (Table S5). In the case of model 6, interannual variation, which was accounted for by including year as a factor, increased the pseudo *R* ^2^ of the model compared to the univariate relationship with only age (Table 5; Table S6). This indicates that there is variation explained by year with regard to reproductive success, with some years having higher probabilities of individuals producing an offspring than others (Figure S5). For both sexes, the probability of successful reproduction was highest in 2018 in all models selected. Higher growth rate percentile increased the probability of producing an offspring in females (Table S7) after accounting for interannual variation. Faster‐growing females had the highest probability of producing an offspring, while females in 2018 had a higher probability of producing an offspring overall (Figure S6).

**TABLE 5:** Median model diagnostics for models that were selected during the model selection procedure.

Model predictors	Model selected (*n*)	Sex	*R* ^2^	Δ_BIC_	BIC_w_	*n*
~Growth Rate + Year	1000	F	0.073	3.18	0.774	957
~Year	546	M	0.568	0.805	0.508	944
~Age + Year	454	M	0.068	0.645	0.483	944

Abbreviations: BIC_w_, the median model weight in iterations in which the model was chosen as the most likely model; *R* ^2^, Nagelkerkes *R* ^2^; Δ_BIC_, the difference in BIC between the selected model and the next lowest BIC value among the candidate models.

## 4 DISCUSSION

In Escanaba Lake, walleye maternal traits such as TL and growth rate percentile were weakly, but positively related to the probability that an offspring was detected, which we used as a measure of reproductive success. Maternal effects have been broadly related to female length in many fishes (refer to Hixon et al., 2014 for examples), including walleye (e.g., Feiner et al., 2016; Johnston, 1997; Johnston & Leggett, 2002). Consequently, our modeling results aligned with previous findings and support the hypothesis that longer females may be more likely to contribute offspring to a specific year class, perhaps by producing more eggs (Shaw et al., 2018) or larger eggs (Wang et al., 2012). In a previous study testing for maternal effects in walleye, Johnston and Leggett (2002) found that growth rate in females had a negative effect on egg size but explained relatively little additional variation (<0.5%) in egg size when included. To our knowledge, our study may be the first to demonstrate a relationship between age‐adjusted size (growth percentile) in female walleye and the probability of producing an offspring. However, even in the presence of weak maternal effects on the probability of producing an offspring, we found that the total number of offspring detected for a TL interval generally reflected the observed frequency of female walleye for the interval, meaning that we did not observe disproportionate contributions from longer females. Moreover, the number of offspring detected was highest for fish between 450 and 500 mm, which is relatively small for sexually mature female walleye (Bozek et al., 2011), but was the most frequent size class in the population. This size distribution of females in Escanaba is partially a product of the harvest restrictions that were in place at the time of the study, which has caused slow growth due to density dependence. Similarly, Lavin et al. (2021) reported that smaller female coral grouper *Plectropomus maculatus* contributed more to reproduction through sheer numbers, even though large individuals were more likely to reproduce, and Andersen et al. (2019) had similar conclusions based on simulated reproductive outputs of Atlantic Cod *Gadus morhua*. Although we detected a positive correlation between TL and reproductive success for female walleye in Escanaba Lake, this did not appear to result in disproportionate contributions to recruitment from longer females in relation to their relative abundance. In the absence of unequivocal relationships between female traits such as length and growth rate and reproductive success, egg characteristics such as fatty acid composition may determine reproductive success (Johnston et al., 2021) and may be coupled more to environmental factors such as productivity than female traits (Wang et al., 2012).

Our results indicated that male contributions to recruitment may be more random than females given there were no relationships between male reproductive success and TL, age, or growth rate. Documented examples of paternal effects are far less numerous for wild populations, except for species showing paired mating (Rowe & Hutchings, 2003; Wiegmann & Baylis, 1995; Zuckerman et al., 2014) or parental care (Green & McCormick, 2005; Kolm, 2002). Our results indicate that the attributes of male walleye measured in this study and size structure of spawning males may not explain parental contributions to recruitment whereas maintaining an adequate number of males relative to female abundance may increase recruitment potential. Like females, the number of offspring produced by males was nearly proportional to the number of males at a given length.

Walleye spawn annually in large aggregations (Bozek et al., 2011), but little is known about behavior associated with individual spawning bouts. The average number of mates observed for female walleye in Escanaba Lake (1.44) indicated that typically only 1–2 males successfully fertilize eggs that result in offspring for an individual female. This could mean that the average number of males involved in an individual spawning bout for an individual female is ≤2 fish or that many males are involved, but only 1–2 typically fertilize eggs. Similarly, the average number of mates per male (1.28) also indicated that many spawning bouts that resulted in an offspring were often the result of paired mating between one male and one female, although it is unknown if males attempted spawning with more than 1–2 females. Estimates of walleye sex ratios for northern Wisconsin walleye populations are obtained during or shortly after spawning and may be male‐biased because males arrive earlier to spawning locations and stay longer than females (Bade et al., 2019), making it difficult to ascertain whether skewed sex ratios might limit recruitment potential in some populations.

Our results were consistent with many previous studies reporting that annual variation in walleye recruitment is often strongly related to environmental conditions (Beard et al., 2003; DeBoer et al., 2013; Honsey et al., 2020). In 2018, a substantially greater number of parents produced offspring detected in our sampling and the estimate of age‐0 walleye abundance (7381) was greater than observed in 2017, 2019, and 2020 (590–844 age‐0 walleye) despite the fact that adult population estimates were relatively consistent over the same period (range = 2008–2608 adult walleye) (Sass et al., 2021). Our estimate of N_s_ in 2018 was considerably greater than other years, while the number of offspring produced per individual was relatively similar across all 4 years of the study, although more variable in 2018. Collectively, these observations indicate that more parents produced offspring in 2018 than in other years rather than a scenario where a similar number of successful parents produced more offspring than in other years. Therefore, high productivity years may have an outsized influence in boosting population‐wide genetic and phenotypic diversity. Based on climatological data for Escanaba Lake estimated using the General Lakes Model (GLM) model (Winslow et al., 2017), spring 2018 had a greater warming rate than any spring during 1980–2019, the years in which the data were available. Additionally, the mean summer temperature in 2018 was in the 97th percentile, and the ice duration was relatively long (89^th^ percentile). A recent review of factors affecting walleye and yellow perch populations found some support for cold winters followed by warm summers benefitting walleye recruitment (Hansen et al., 2022). Specifically, studies conducted on lakes in Minnesota and Nebraska found that warm winters with early ice‐out dates were detrimental to walleye recruitment (e.g., DeBoer et al., 2013; Schneider et al., 2010). Similarly, studies have found that rapidly warming springs were beneficial to walleye recruitment (e.g., DeBoer et al., 2013; Graeb et al., 2010; Zhao et al., 2013), although studies focused on walleye populations in northern Wisconsin have shown mixed results with regard to spring conditions (Hansen et al., 2015, 2017). For walleye in Escanaba Lake, Hansen et al. (1998) reported that variation in May water temperature was an important factor explaining variation in recruitment. Shaw et al. (2018) also found that the variation in spring temperatures following ice out was important, as the relationship between spring temperature variability and recruitment was negative.

Walleye recruitment declines have occurred in some lakes in the upper midwestern USA, (Hansen et al., 2018, 2020; Honsey et al., 2020), and loss of recruitment may continue to occur in relation to climate change (Hansen et al., 2018; Honsey et al., 2020). Consequently, management strategies that maximize walleye recruitment potential could help in offsetting these declines. Our results indicate that implementing harvest restrictions to increase size structure of adult walleye may have only small effects on recruitment potential, especially if the improvements in size structure are minimal, which has been the case in several previous studies (Fayram et al., 2001; Isermann, 2007; Koupal et al., 2015) including Escanaba Lake (Haglund et al., 2016). However, implementation of length‐based harvest restrictions may increase walleye recruitment potential in some lakes by increasing the probability that females are provided at least one opportunity to contribute to recruitment before they are harvested. As of 2023, the minimum TL limit for walleye harvest in most northern Wisconsin lakes is 381 mm (the regulation also includes a protected slot from 508 to 609 mm and a limit of one fish >609 mm, refer to https://dnr.wisconsin.gov/topic/fishing/regulations). This regulation was enacted in part because harvest had been dominated by fish <381 mm and many females are not mature at this length (Fayram et al., 2001), meaning they were removed before spawning at least once, which could lead to recruitment overfishing. Hence, enacting a 381‐mm minimum length theoretically provided a greater probability that female walleye could spawn at least once before they could potentially be harvested. However, our results from Escanaba Lake indicate that if female walleye are harvested before they attain 400 mm TL, they may have had the opportunity to spawn at least once, but they have a low probability of contributing to recruitment. In lakes with maturity schedules similar to or slower than Escanaba Lake, increasing the minimum TL for harvest to a TL ≥400 mm could increase the probability that female walleye have one opportunity to contribute to recruitment prior to becoming vulnerable to harvest. Moreover, our results indicated that faster‐growing female walleye in Escanaba Lake were more likely to contribute to recruitment and these fish may be exposed to harvest earlier under a minimum length limit than slower‐growing fish. Therefore, increasing the minimum length limit for harvest to a TL > 400 mm may also provide a greater opportunity for fast‐growing female fish to contribute to recruitment at least once before they are vulnerable to harvest.

We note that increasing walleye recruitment potential may not result in increased recruitment. Two previous studies have shown that increasing minimum length limits did not result in increased walleye recruitment (Haglund et al., 2016; Stone & Lott, 2002), but Haglund et al. (2016) reported that variation in recruitment was lower after a minimum TL limit of 710 mm was enacted on Escanaba Lake. Additionally, increasing the number of age‐0 walleye that may be produced in a system may not translate to higher recruitment at later life stages if density‐dependent mortality factors regulate survival to these later stages (Rose et al., 1999).

Stocking is the primary tool used to support walleye populations where natural recruitment is not sufficient to meet fishery objectives (Kerr, 2011; Raabe et al., 2020), and increased stocking was implemented in northern Wisconsin in response to walleye recruitment declines (Elwer et al., 2023; Lawson et al., 2022). Additionally, stocking represents the sole source of recruitment for many walleye fisheries in North America (Kerr, 2011). If walleye recruitment continues to decline in populations where fisheries were historically supported by natural recruitment, demand for stocking may increase. Our results from Escanaba Lake have implications for stocking walleye because wild broodstock are typically used as the source of gametes for production of stocked fish. If parental traits also influence hatching success and survival of fish before and after stocking, selection of broodstock and hatchery protocols could influence stocking success. The WDNR attempts to select walleye broodstock at random with no preference for TL or other attributes other than the fish appearing to be healthy. One of the goals of this process is to meet criteria for genetic diversity that are based on phenotypic diversity observed in source populations (Bootsma et al., 2021; Hammen & Sloss, 2019). Theoretically, based on our results from Escanaba Lake, selecting for longer, faster‐growing female fish may provide a slight increase the probability that offspring survive to their first fall either in the wild (if fish are stocked as fry) or in hatchery ponds used to rear fish before they are stocked. However, imposing artificial selection through broodstock selectivity could reduce the genetic diversity of stocked fish. Additionally, offspring survival through early life history stages in a hatchery setting may not translate to success once stocked due to consequences of hatchery‐induced selection (Araki et al., 2008; Blouin et al., 2021; Lynch & O'Hely, 2001).

The number of mates we observed for adult walleye in Escanaba Lake also has potential implications for hatchery protocols. The WDNR typically uses 5–6 males to fertilize one pan of eggs, which can be from only one female. This formula can limit the number of potential parental crosses that can be accomplished if availability of males is a low. Our results indicate that using 1–2 males per female could better simulate natural conditions as observed in Escanaba Lake. In situations where availability of males is a limiting factor in the propagation process, using fewer males per female could result in less sampling effort needed to collect additional males or increase the number of crosses that could be made in the hatchery, which could increase the genetic diversity of stocked fish.

Despite the relatively intensive sampling that occurs on Escanaba Lake for both adult and juvenile walleye, failure to account for all potential parents and offspring results in some uncertainty associated with our findings. Previous efforts to reconstruct genetic pedigrees in fish often occur in populations where there are either a small number of potential parents (e.g., Sard et al., 2020) or in fisheries where adults are confined in a small area such as the tail race of a dam or are territorial (e.g., Lavin et al., 2021; Sard et al., 2015). In the context of this study, increasing spring sampling effort to capture more adult walleye could aid in determining specific information within a single population, such as population demographics (e.g., sex‐ratio, age‐at‐maturity, mortality) and physical characteristics of potential parents (e.g., length, weight, age). Alternatively, increasing sampling effort in the fall of age‐0 walleye can yield more information regarding the size of the breeding population, number of mates per individual, and reproductive skew without prior knowledge of the parents due to the potential use of sibship reconstruction. To this end, much of the information regarding recruitment dynamics can be obtained solely from age‐0 offspring through sibship rather than a parentage‐based approach. This can alleviate the need for the labor‐intensive collection of adults throughout spring spawning in the case of walleye while providing information about the effective breeding population size, number of offspring per individual, number of mates per individual, inbreeding coefficient, non‐random mating, and reproductive skew.

Protecting larger females that may produce disproportionately more offspring is a paradigm of fisheries management in marine and freshwater fisheries (Gwinn et al., 2015; Hixon et al., 2014). However, directly evaluating this hypothesis is nearly impossible without large‐scale pedigrees, which are extremely rare for broadcast spawners. We are only aware of three large‐scale pedigrees in broadcast spawners, our system, coral grouper (*Plectropomus maculatus*) in Australia (Lavin et al., 2021), and a long‐running pedigree for lake sturgeon (*Acipenser fulvescens*) from the Black River, Michigan, USA (Duong et al., 2023). Results from all three studies indicate that while larger females may contribute more offspring on a per capita basis, the magnitude of the effect may be modest and largely offset by the greater abundances of individuals of smaller sizes whose growth potential may be unrealized. Moreover, the commonly observed positive relationship between fish body size and egg size (hence quality) does not necessarily result in a greater number of surviving offspring per gonadal volume of larger individuals (Koenigbauer & Höök, 2023), consequently diminishing the potential selective pressure associated with removal of large female. In summary, our results and those of Duong et al. (2023) and Lavin et al. (2021) challenge the paradigm that large females produce disproportionally more offspring and are integral to the reproductive potential of fish populations. However, observed variation in the relative reproductive success of large individuals among these three studies justifies further investigation. Increasing efficiencies in genotyping could enable additional large‐scale parentage analyses, providing a means to test this hypothesis more robustly and identify additional factors that influence relationships between fish traits and reproductive success.

## CONFLICT OF INTEREST STATEMENT

The authors have no conflicts of interest to disclose.

## Supporting information


Appendix S1

Table S1

## Data Availability

Coauthor and data author, Robert Davis, has made the phenotypic data and genotypes for walleye in this study which are available via Dryad. Questions about the data can be directed to the data author, Robert Davis at robert.davis.bd@gmail.com.
