# Supplementary material for: Demographic patterns of walleye (Sander vitreus) reproductive success in a Wisconsin population
Source: Evol Appl. 2024 Mar 10;17(3):e13665. doi: 10.1111/eva.13665 (PMC10925830; doi:10.1111/eva.13665)
Supplement: Supplementary file 1 — Appendix S1 [file EVA-17-e13665-s002.docx]

**Supplemental Information**

Demographic patterns of walleye (*Sander vitreus*) reproductive success in a Wisconsin Population

Robert P. Davis^1^*, Levi M. Simmons^1^, Stephanie L. Shaw^2^, Greg G. Sass^2^, Nicholas M. Sard^3^, Daniel A. Isermann^4^, Wesley A. Larson^5^ Jared J. Homola^4^

^1^Wisconsin Cooperative Fishery Research Unit, University of Wisconsin-Stevens Point, Stevens Point, Wisconsin, USA

^2^Office of Applied Science, Wisconsin Department of Natural Resources, Escanaba Lake Research Station, Boulder Junction, Wisconsin, USA

^3^Department of Biological Sciences, State University of New York-Oswego, Oswego, NY USA

^4^U.S. Geological Survey, Wisconsin Cooperative Fishery Research Unit, University of Wisconsin-Stevens Point, Stevens Point, Wisconsin, USA

^5^National Marine Fisheries Service, Alaska Fisheries Science Center, Auke Bay Laboratories, National Oceanic and Atmospheric Administration, Juneau, Alaska, USA

** Corresponding Author*, email: rdavis@northland.edu

Any use of trade, firm, or product names is for descriptive purposes only and does not imply endorsement by the U.S. Government.

*Simulation Study*

**Methods**

Simulations were used to evaluate how several factors associated with the reproductive ecology of walleye *Sander vitreus* affect estimates in the number of spawning individuals (N_s_). Specifically, R scripts used in Sard et al. (2020) were modified to consider how sex ratio (SR: 1,2,3; male to female), lambda – i.e., mean and variance in the number of mates, (λ = 2, 3, 4), adult population size (N = 2000 or 3000), the proportion of the adult population that successfully reproduce (P_N_ = 0.2, 0.3, or 0.4), and non-random survival of offspring affected estimates of N_s­_. That is, little is known about reproductive ecology in the system, so we evaluated a range of possible scenarios to consider how sensitive N_s_ values were to varying the above parameters. The Escanaba system is well studied, and accordingly information from ongoing research helped inform model parameterization. First, the size of the adult population has been estimated using mark-recapture methods; estimates from 2017-2022 (range: 2008-2862) were used to set the lower and upper limits evaluated of N in simulations. During adult sampling, the SR was also estimated from 2017-2022 (range: 1.15-3.95), which again informed parameterization of the model. While the Escanaba system is well studied, there is no published information to inform what to set λ or P_N_, which is why we evaluated a range of values for each parameter to consider how changed to each values affect estimates of N_s_.

Each simulation consisted of constructing a breeding matrix, where columns represent females and rows represent males. The number of columns and rows in the matrix were determined based on the specific values for N, P_N_, and SR. Among breeding adults, mates were identified based on λ. Among females, we used the estimated age distribution and age-specific fecundity values to determine the specific values of fertilized eggs for each female. That is, each female was randomly assigned an age based on a weighted probability distribution. Using a female’s age and a published equation (Sern 1982, Table 4), fecundity was estimated. Fertilized eggs for each female were uniformly distributed among mates. The simulation model used here has been shown to be insensitive to variation in fitness values among males (Sard et al. 2020). Finally, survival among fertilized eggs is likely non-uniform, and thus, we evaluated the effect of variable survival among clutches by randomly down-sampling the number of fertilized eggs per female based on estimates of survival rates (mean ± 1 Standard Deviation (SD), 9.96E-4 ± 1.033E-3, Shaw et al. 2018). We compared simulations with and without down sampling to determine how much variable survival rates affect estimates of N_s_. Finally, once the breeding matrix was assembled, we randomly subsampled all offspring produced in a breeding season to a sample of 200 offspring. The parent-offspring relationships within the sampled pedigree were used to estimate N_S_. In total, we conducted 100 independent simulations for each unique combination of parameters described above (n = 108 combinations). Across the 10,800 N_s_ values estimated, we compared each to known N_s_ values by calculating bias ($\frac{\sum_{i=1}^{n} \frac{\hat{N}_{s}}{N_{s}}}{n}$). To quantify the effect of each parameter individually, we summarized differences in bias estimates among specific values within a parameter value. For instance, to determine the effect of changing a sex ratio from 1:1 to 2:1, we subtracted estimates of bias for 2:1 from 1:1 simulations, while holding all other parameters constant. We then summarized the mean ± 1 SD among the differences calculated. While there is much uncertainty in the system, the model described above enables an improved understanding of how adult and age-0 fish sampling can affect estimates of N_S_.

**Results**

Simulations demonstrated that N_s_ estimates are expected to be biased low. Generally, when 200 offspring were sampled, increasing the proportion of the population that successfully bred or increasing sex ratio decreased N_s_ estimates. In contrast, increasing λ marginally affected estimates. Generally, increasing λ, across parameter space evaluated, reduced N_s_ by less than 3% (Supplemental Table 1). Deviations from 1:1 sex ratios had a larger effect, but all were less than 10% reductions in N_S_ estimates. Accounting for variable survival among clutches reduced N_s_ estimates by an additional 10% (Supplemental Table 1). Importantly, simulations helped to interpret N_S_ among years by contrasting extreme scenarios evaluated. For instance, when comparing a scenario when 20% of a population of 2000 individuals bred, and the sex ratio is 1:1 and λ = 4, the expected bias for N_S_ estimates was a 22% reduction compared to known values. Compared to a scenario when 40% of the 2000 adults bred, at a sex ratio of 3:1 and λ = 2, the expected reduction in N_s_ estimates was 45%, compared to known N_s_ values. Thus, among years evaluated, we expect that a 23% change in N_s_ estimates could be accounted for by natural variation in the reproductive ecology of walleye in this system.

Supplementary Table 1. Summary of the mean (± 1 SD) change in $\frac{\hat{N}_{s}}{N_{s}}$ for specific parameters modeled, while holding other parameters constant, which were interpreted as metric describing how sensitive results were to changes in assumptions in the model. Whether survival was modeled or not is indicated by the Survival Filter column. The parameter column identifies what parameter (sex ratio or lambda) was evaluated to determine how $\frac{\hat{N}_{s}}{N_{s}}$ changed from one value modeled to another when the total population size of 2000 or 3000. For example, "Lambda 2 --> 3" when survival was modelled (i.e., Survival Filter = Yes), describes that a 5.4% ± 1.5% or 5.8% ± 2.0% change in $\frac{\hat{N}_{s}}{N_{s}}$ were expected when 2000 or 3000 adults, respectively, existed in the system, when the sex ratio changed from 1:1 to 2:1 (males:females).

|  |  | **N = 2000** | | **N = 3000** | |
| --- | --- | --- | --- | --- | --- |
| **Survival Filter** | **Parameter** | **Mean** | **SD** | **Mean** | **SD** |
| Yes | Lambda 2 --> 3 | 0.023 | 0.010 | 0.018 | 0.015 |
|  | Lambda 3 --> 4 | 0.016 | 0.011 | 0.020 | 0.014 |
|  | Sex ratio 1 --> 2 | 0.054 | 0.015 | 0.058 | 0.020 |
|  | Sex ratio 2 --> 3 | 0.050 | 0.011 | 0.053 | 0.013 |
| No | Lambda 2 --> 3 | 0.012 | 0.018 | 0.017 | 0.016 |
|  | Lambda 3 --> 4 | 0.011 | 0.020 | 0.008 | 0.018 |
|  | Sex ratio 1 --> 2 | 0.076 | 0.020 | 0.085 | 0.020 |
|  | Sex ratio 2 --> 3 | 0.065 | 0.012 | 0.082 | 0.019 |
|  | Survival vs no survival filter | 0.091 | 0.022 | 0.090 | 0.029 |

*Supplemental Figures and Tables*

*Tables*

Supplementary Table 2. The distribution of total offspring (n) assigned to individual parents in in this study.

| N offspring | n |
| --- | --- |
| 0 | 199 |
| 1 | 197 |
| 2 | 59 |
| 3 | 37 |
| 4 | 10 |
| 5 | 9 |
| 6 | 3 |
| 7 | 2 |
| 8 | 1 |
| 9 | 1 |
| 10 | 1 |
| 12 | 1 |

Supplementary Table 3. Number of offspring detected per individual in each year of this study. Sd= standard deviation

| Year | Female | | Male | |
| --- | --- | --- | --- | --- |
|  | Mean | Sd | Mean | Sd |
| 2017 | 1.27 | 0.49 | 1.24 | 0.62 |
| 2018 | 1.76 | 1.32 | 1.43 | 0.81 |
| 2019 | 1.32 | 1.03 | 1.11 | 0.32 |
| 2020 | 1.31 | 0.78 | 1.18 | 0.62 |

Supplementary Table 4. Summary statistics for adults sampled for this study.

| Year | Sex | Mean Length (mm) | std. dev | max | min | Mean age (year) | std. dev | max | min | n |
| --- | --- | --- | --- | --- | --- | --- | --- | --- | --- | --- |
| 2017 | Female | 492.6 | 54.1 | 737 | 406 | 9.8 | 2.6 | 17 | 5 | 67 |
|  | Male | 389.8 | 41.2 | 467 | 287 | 6.5 | 2.4 | 13 | 4 | 96 |
| 2018 | Female | 473.4 | 49.2 | 699 | 386 | 8.0 | 2.7 | 19 | 5 | 112 |
|  | Male | 386.5 | 38.0 | 465 | 297 | 5.7 | 2.7 | 16 | 2 | 99 |
| 2019 | Female | 447.5 | 50.5 | 579 | 353 | 7.5 | 2.8 | 17 | 3 | 79 |
|  | Male | 400.3 | 36.9 | 478 | 302 | 7.4 | 3.4 | 15 | 3 | 63 |

Supplementary Table 5. Model parameters for Model 3 for males (probability of reproducing an offspring ~ Year). SE = standard error, OR_slope_ = odds ratio of the slope, and CI_OR_ = 95% confidence interval of the odds ratio of the slope.

| Predictor | Coefficient | SE | OR | CI_OR_ |
| --- | --- | --- | --- | --- |
| 2017 | -1.77 | 0.194 | 0.170 | 0.140 - 0.207 |
| 2018 | -0.56 | 0.133 | 0.571 | 0.500 - 0.652 |
| 2019 | -1.49 | 0.169 | 0.225 | 0.190 - 0.267 |
| 2020 | -1.07 | 0.161 | 0.343 | 0.292 - 0.403 |

Supplementary Table 6. Model parameters for Model 6 for males (probability of reproducing an offspring ~ Age + Year). SE = standard error, OR_slope_ = odds ratio of the slope, and CI_ORslope_ = 95% confidence interval of the odds ratio of the slope

| Predictor | Coefficient | SE | OR_slope_ | CI_ORSlope_ | |
| --- | --- | --- | --- | --- | --- |
| Intercept | -1.238 | 0.265 |  |  |  |
| 2018 | 1.241 | 0.237 |  |  |  |
| 2019 | 0.369 | 0.260 |  |  |  |
| 2020 | 0.873 | 0.261 |  |  |  |
| Age (slope) | -0.080 | 0.03 | 0.923 | 0.896 - 0.951 | |

SI Table 7. Model parameters for Model 7 for females (probability of reproducing an offspring ~ Growth Rate + Year). SE = standard error, OR_slope_ = odds ratio of the slope, and CI_ORslope_ = 95% confidence interval of the odds ratio of the slope.

| Predictor | Coefficient | SE | OR_slope_ | CI_ORSlope_ | |
| --- | --- | --- | --- | --- | --- |
| Int | -1.973 | 0.231 |  |  |  |
| 2018 | 0.937 | 0.216 |  |  |  |
| 2019 | -0.312 | 0.259 |  |  |  |
| 2020 | 0.023 | 0.253 |  |  |  |
| Growth Rate (slope) | 0.769 | 0.291 | 2.158 | 1.613 - 2.886 | |

*Figures*


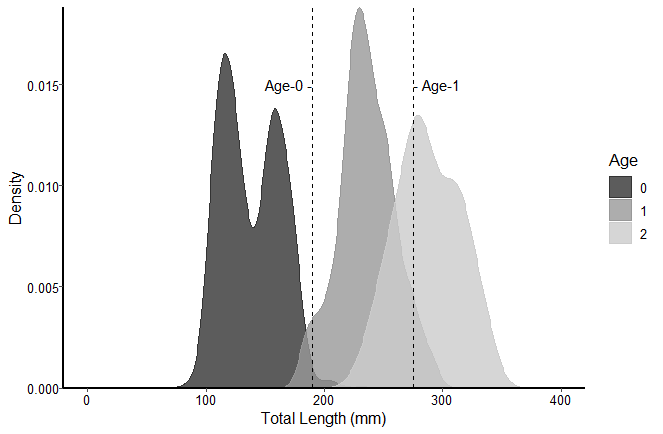


Supplementary Figure 1. Age-length frequency for young of the year and juvenile walleye in Escanaba Lake between 2017 and 2020. Fish included in this figure include individuals sampled during fall electrofishing surveys (age-0 and age-1) and individuals from spring fyke net surveys (age-2). Vertical dashed lines represent upper limit length cutoffs that were used to determine age in un-aged individuals, with the age-0 maximum cutoff at 190 mm and the age-1 maximum cutoff at 275 mm.


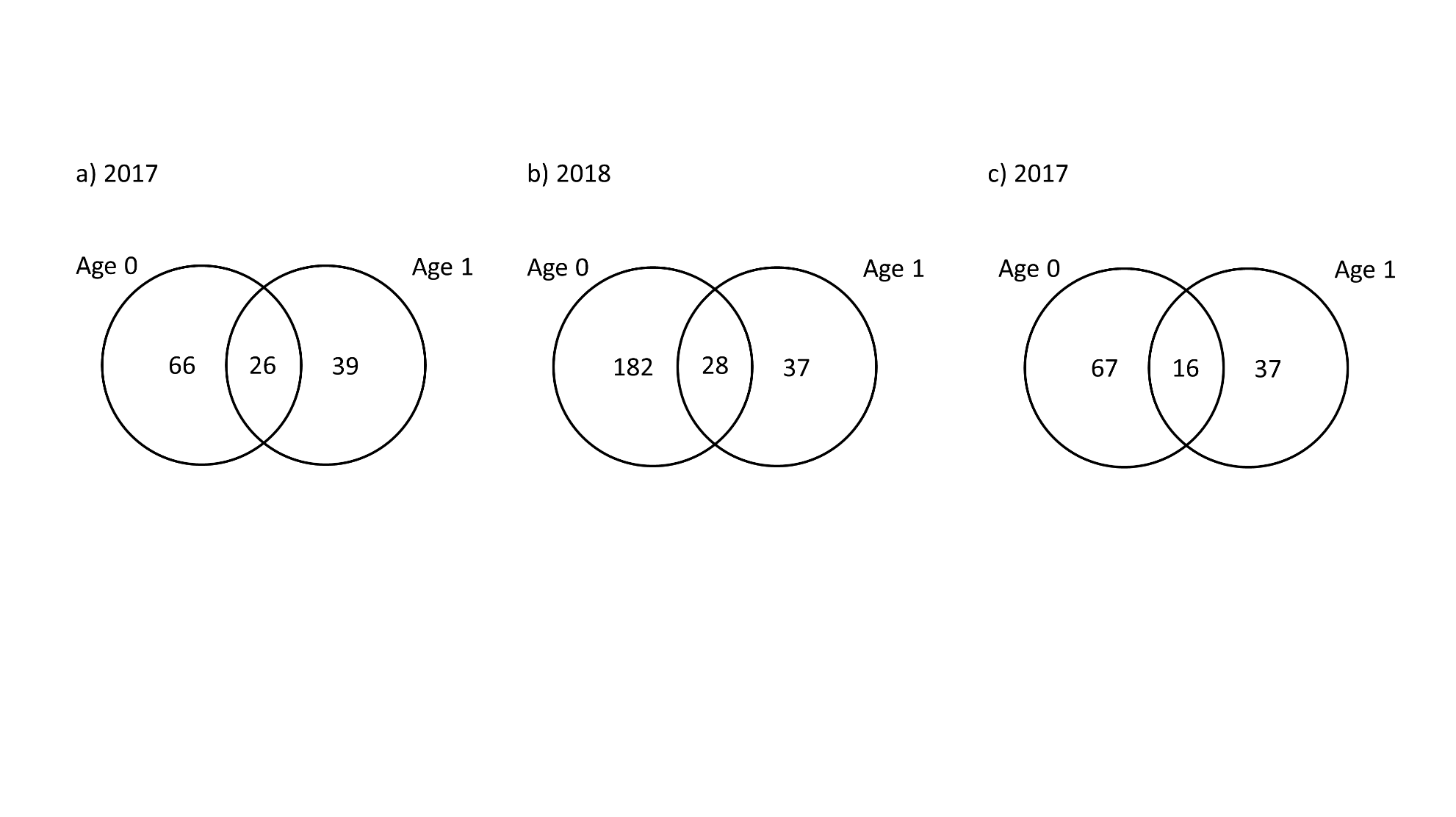


c) 2019

Supplementary Figure 2. Venn diagram of parents detected among age-0 and age-1 the following year within the same cohort. Parent-offspring relationships were compared among age-0 and age-1 walleye for each cohort where the data were available for two consecutive years.


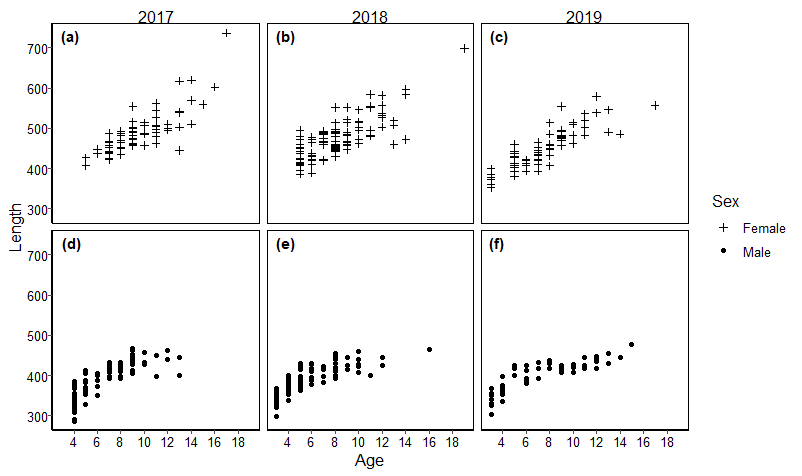


Supplementary Figure 3. Length at age plots for female and male walleye sampled for this study by year.


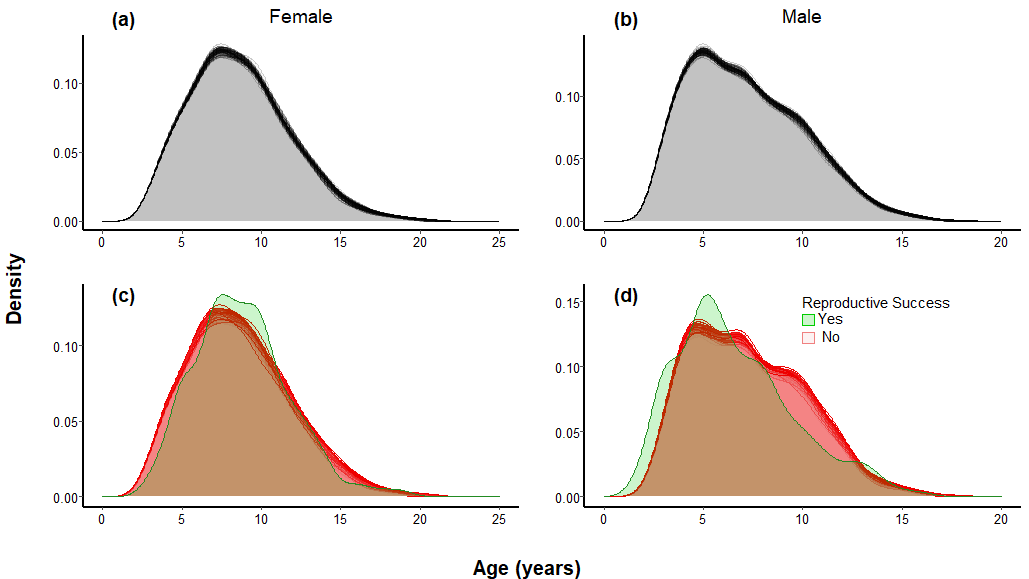


Supplementary Figure 4. Density plots of simulated age distributions (n=100 iterations) of the overall (4a and 4b) potential pool of parents sampled for this project and the distribution of successful parents (green) and simulated distribution of unsuccessful (red, n = 100 each) parents for female (4c) and male (4d) parents in Escanaba Lake 2017-2019.


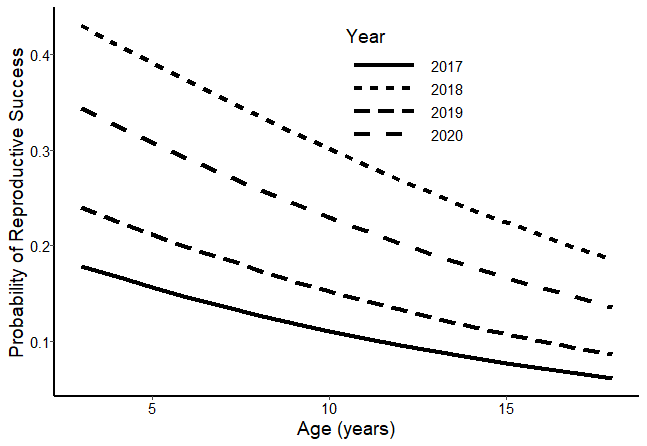


Supplementary Figure 5. Logistic Regression of Model 6 (probability of producing an offspring ~ Age + year ) for male walleye in Escanaba Lake.


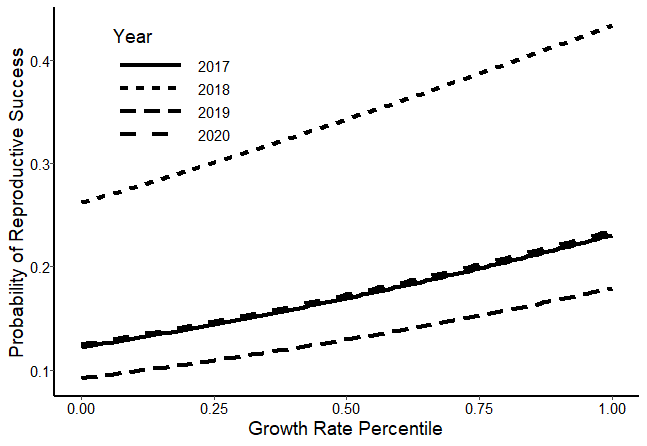


Supplementary Figure 6. Logistic Regression of Model 7 (probability of producing an offspring ~ Growth Rate + Year) for female walleye Escanaba Lake in the years 2017 -2020.

References

Sard NM, Smith SR, Homola JJ, Kanefsky J, Bravener G, Adams JV, Holbrook CM, Hrodey PJ, Tallon K, Scribner KT. 2020. Rapture (rad capture) panel facilitates analyses characterizing sea lamprey reproductive ecology and movement dynamics. Ecology and Evolution. 10(3):1469-1488.

Serns SL. 1982. Walleye fecundity, potential egg deposition, and survival from egg to fall young of year in Escanaba Lake, Wisconsin, 1979-1981. North American Journal of Fisheries Management. 2(4):388-394.

Shaw, S. L., G. G. Sass, and J. A. VanDeHey. 2018. Maternal effects better predict walleye recruitment in Escanaba Lake, Wisconsin, 1957-2015: implications for regulations. Canadian Journal of Fisheries and Aquatic Sciences 75(12):2320-2331.
