# Supplementary material for: Demographic patterns of walleye (Sander vitreus) reproductive success in a Wisconsin population
Source: Evol Appl. 2024 Mar 10;17(3):e13665. doi: 10.1111/eva.13665 (PMC10925830; doi:10.1111/eva.13665)
Supplement: Supplementary file 2 — Table S1 [file EVA-17-e13665-s001.docx]

Supplementary Table 1. Summary of the mean (± 1 SD) change in $\frac{\hat{N}_{s}}{N_{s}}$ for specific parameters modeled, while holding other parameters constant, which were interpreted as metric describing how sensitive results were to changes in assumptions in the model. Whether survival was modeled or not is indicated by the Survival Filter column. The parameter column identifies what parameter (sex ratio or lambda) was evaluated to determine how $\frac{\hat{N}_{s}}{N_{s}}$ changed from one value modeled to another when the total population size of 2000 or 3000. For example, "Lambda 2 --> 3" when survival was modelled (i.e., Survival Filter = Yes), describes that a 5.4% ± 1.5% or 5.8% ± 2.0% change in $\frac{\hat{N}_{s}}{N_{s}}$ were expected when 2000 or 3000 adults, respectively, existed in the system, when the sex ratio changed from 1:1 to 2:1 (males:females).
